# Design and Development of a Nearable Wireless System to Control Indoor Air Quality and Indoor Lighting Quality

**DOI:** 10.3390/s17051021

**Published:** 2017-05-04

**Authors:** Francesco Salamone, Lorenzo Belussi, Ludovico Danza, Theodore Galanos, Matteo Ghellere, Italo Meroni

**Affiliations:** 1 ITC-CNR, Construction Technologies Institute—National Research Council of Italy, Via Lombardia 49-20098 San Giuliano M.se, Italy; belussi@itc.cnr.it (L.B.); danza@itc.cnr.it (L.D.); ghellere@itc.cnr.it (M.G.); meroni@itc.cnr.it (I.M.); 2 NEAPOLI SDN BHD 894646-M Environmental Design and Engineering, D-8-5, Megan Avenue 1, 189 Jalan Tun Razak, 50400 Kuala Lumpur, Malaysia; theodore@neapoli.com.my

**Keywords:** open source, Arduino, do-it-yourself (DIY), Internet of things (IoT), control system, environmental monitoring system, building automation, indoor thermal comfort quality, indoor environmental quality, indoor air quality, indoor lighting quality, energy saving

## Abstract

The article describes the results of the project “open source smart lamp” aimed at designing and developing a smart object able to manage and control the indoor environmental quality (IEQ) of the built environment. A first version of this smart object, built following a do-it-yourself (DIY) approach using a microcontroller, an integrated temperature and relative humidity sensor, and techniques of additive manufacturing, allows the adjustment of the indoor thermal comfort quality (ICQ), by interacting directly with the air conditioner. As is well known, the IEQ is a holistic concept including indoor air quality (IAQ), indoor lighting quality (ILQ) and acoustic comfort, besides thermal comfort. The upgrade of the smart lamp bridges the gap of the first version of the device providing the possibility of interaction with the air exchange unit and lighting system in order to get an overview of the potential of a nearable device in the management of the IEQ. The upgraded version was tested in a real office equipped with mechanical ventilation and an air conditioning system. This office was occupied by four workers. The experiment is compared with a baseline scenario and the results show how the application of the nearable device effectively optimizes both IAQ and ILQ.

## 1. Introduction

The term “nearable” (or nearable technology), used for the first time in 2014 as part of a marketing campaign, is now used to uniquely identify the idea of smart objects that can be equipped with a variety of sensors and can work as transmitters to broadcast digital data [1]. This technology finds applications in several fields; the present article describes the potential in building automation. In the past years, the stakeholders of the building sector have been mainly involved in the design of new solutions to maximize the performance of technological systems in order to meet the requirements of the zero-energy building (ZEB) concept [2]. Today they are more and more involved in the development of semantic data models [3,4], calculation methodologies [5,6,7], models [8,9] and systems able to acquire, store and mine building data through the connection of building information modeling (BIM) and the Internet of things (IoT) [10,11,12]. The extreme flexibility of the new pervasive technologies [13] allows their application in different fields, like indoor environmental quality (IEQ) management, energy consumption [14] and occupancy behavior monitoring [15].

As is well known, IEQ is a holistic concept including indoor air quality (IAQ), indoor lighting quality (ILQ) and indoor acoustic comfort [16,17], besides indoor climate quality (ICQ). The above considerations are the basis for design and development of the smart lamp [18] implemented by researchers of the Construction Technologies Institute—National Research Council of Italy (ITC-CNR) [19] following the principles of the “maker movement” philosophy and the do-it-yourself (DIY) approach [20,21,22] which is applied more and more in different contexts: from monitoring systems [23,24] to control systems of renewable energy sources (RES) [25,26], to applications in the biomedical field in order to make the equipment less expensive and, consequently, more accessible [27,28]. One such device, built using a microcontroller, and an integrated temperature and relative humidity sensor, as well as some other modules, exploited additive manufacturing (AM) techniques and was applied and tested in an office normally occupied by four workers, equipped with an air conditioning system and naturally ventilated. The analysis of the thermal variables and the energy consumption demonstrated how it is possible to optimally manage the ICQ and the energy performance of the air conditioning system through the use of the smart lamp. The assessment of the other parameters of the IEQ is the natural upgrade of the smart lamp. While in [15] a hybrid detection method using the combination of CO_2_ and light sensors is used to estimate the occupancy completely overcoming the issues related to privacy, in this case, the device, based on a CO_2_ concentration sensor and a photoresistor, provides the possibility to control and optimize the lighting and air quality levels starting up the lighting equipment and the air exchanger. The new version of the smart lamp was tested in the same office by monitoring the environmental variables for 14 days, divided in two periods with different control configurations: the former with manual control (23–29 May 2016) and the latter with automatic control (30 May–5 June 2016).

## 2. Hardware and Software of the Developed Nearable

**Figure 1:**
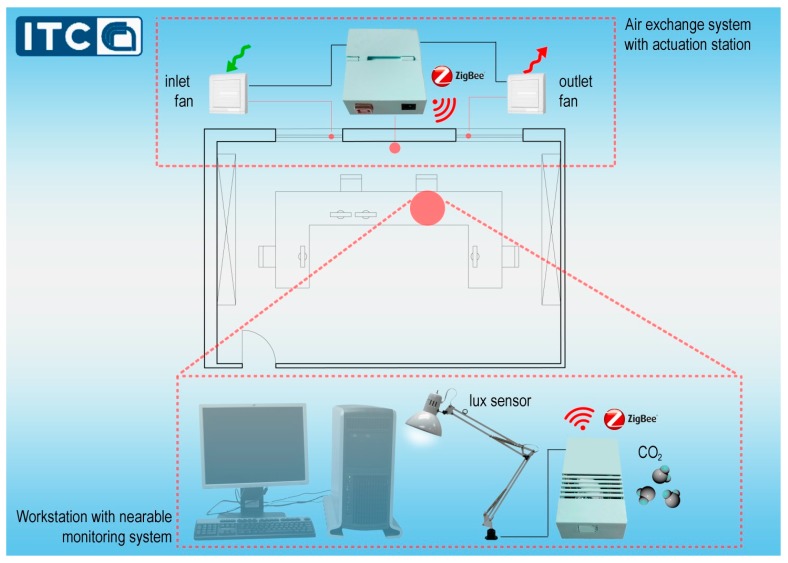
Hardware layout in a real case study.

The hardware and software elements of the system are defined using typical concepts of the DIY philosophy: wireless communication, low cost hardware and 3D printed parts. The system is split in two parts (Figure 1): a monitoring station placed near the workstation (nearable monitoring station) able to assess the exact level of illuminance and air quality, and a wireless receiving station (actuation station) connected to the nearable monitoring station that manages the actuation of both the air exchange system and the lamp. Both parts have small dimensions and weight, in order to be adjustable and to minimize their impact on human activity, especially in workplaces.

### 2.1. Nearable Monitoring and Coordination Station

**Figure 2:**
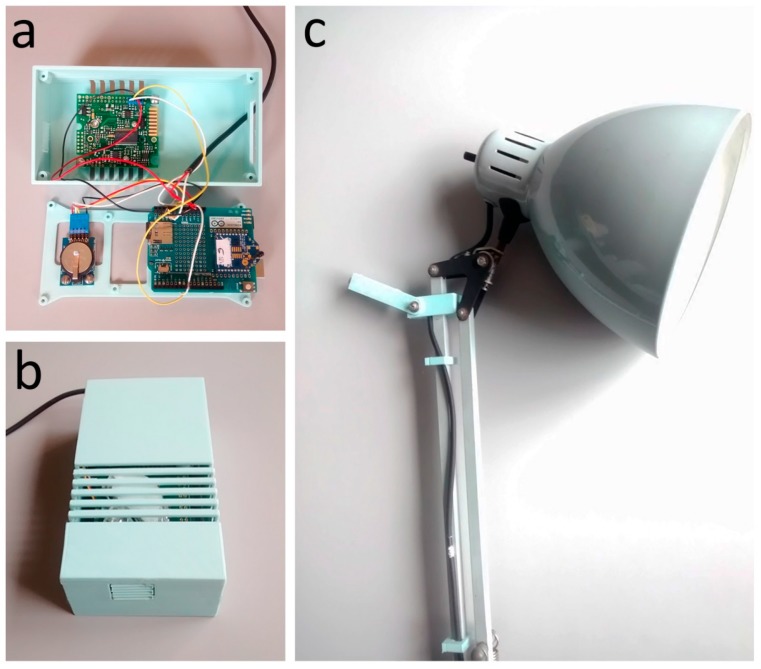
Nearable monitoring and coordination station: (**a**) internal connection; (**b**) external case with the slits for the CO_2_ concentration sensor; (**c**) the lux sensor as mounted on the desk lamp.

The nearable monitoring station (Figure 2), consists of the following elements:

- Arduino UNO r3 with “sandwich connected” wireless shield [29,30],
- XBee S2 module [31];
- Real-time clock (RTC) module based on DS1307 chip [32];
- K30 CO_2_ concentration sensor [33];
- A photoresistor and a 10 kΩ resistor.

**Figure 3:**
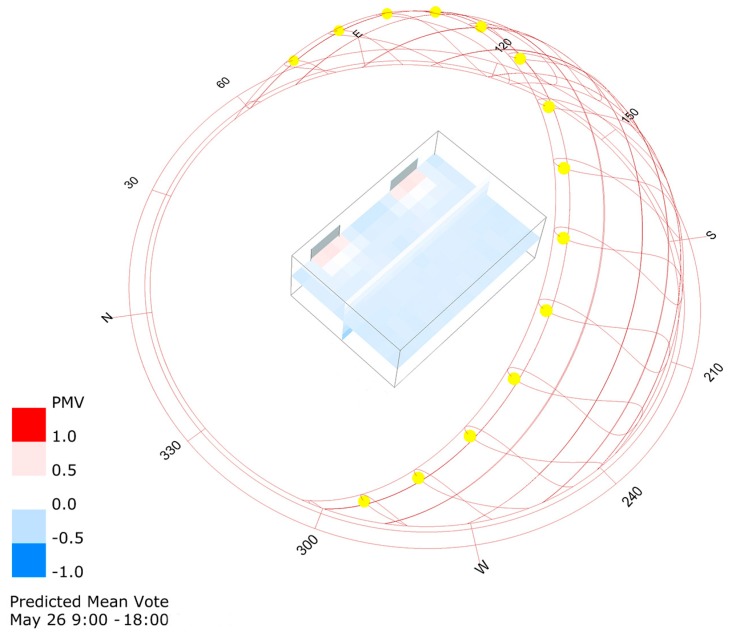
Predicted mean vote (PMV) preliminary analysis for a typical day of the test period: 26 May from 9:00 a.m. to 6:00 p.m.

The basement (Figure 2a) contains the slot of the Arduino UNO r3 board (Smart Project s.r.l, Scarmagno, Italy) and the RTC module. The base is designed to contain also an Arduino MEGA 2560 r3 board in order to consider an all-in-one device that could control the indoor climate quality [18]. The lid (Figure 2b) closes the case, providing a series of slits for the CO_2_ concentration sensors. Its design also includes the housing for the integrated air temperature and relative humidity sensor (Figure 2b) in order to implement the control logic of the ICQ [18]. The photoresistor is connected to the desk lamp by means of a 3D printed adapter (Figure 2c). In this specific case, the desk lamp is located on the desktop at about 0.8 m from the floor. It is equipped with a non-dimmable Light Emitting Diode (LED) bulb type with a power consumption of 10 W, a light output of 810 lm and a natural white light (4200 K). The cost of the hardware (electronics and 3D printed case) of the nearable monitoring station is of about €130, while according to DIY philosophy, the lamp should be composed of recycled material. This amount would be reduced by considering a cheapest CO_2_ concentration sensor because the chosen sensor covers 60% of the total cost. Unlike the previous case, [18] in the upgraded version of the smart lamp, the thermohygrometric sensor is not installed because a preliminary analysis made up combining the EnergyPlus results with Honeybee pre/post processing capabilities (Figure 3) showed an approximated average neutral value for the thermal comfort, expressed by the predicted mean vote (PMV) index. This has suggested the possibility to neglect the control of thermal comfort.

In fact, as may be seen in Figure 3, for 26 May, when the altitude of the sun at 12:00 a.m. is above 65°, the daily average values of the simulated PMV considering the period between 9:00 a.m. to 6:00 p.m. are next to zero. The simulation was carried out considering typical environmental parameters taken from an EnergyPlus weather format (EPW) file of a location closest to the office site (within 10–15 km). The envelope of the office is characterized by two single-pane glass windows; an external wall element [34] consisting of double layer brick masonry not insulated with plaster finishing; the three internal walls considered as an adiabatic element, according to the definition provided by [35], are made of a single layer brick masonry with plaster finishing on each side; the floor is a concrete brick beam structure covered by ceramic pavement over a mortar layer, and is considered as an adiabatic element according to the definition provided by [36]; and the ceiling is the roof of the building [37], characterized by a flat concrete brick beam structure covered by a waterproof membrane without thermal insulation. Further details of the office are shown in the case study paragraph. For the calculation of the PMV index a metabolic rate of 1.1 met and a clothing thermal resistance equal to about 0.6 clo are considered. Indeed, during the test period, no thermal discomfort was reported by the workers.

### 2.2. Receiving Actuation Station

**Figure 4:**
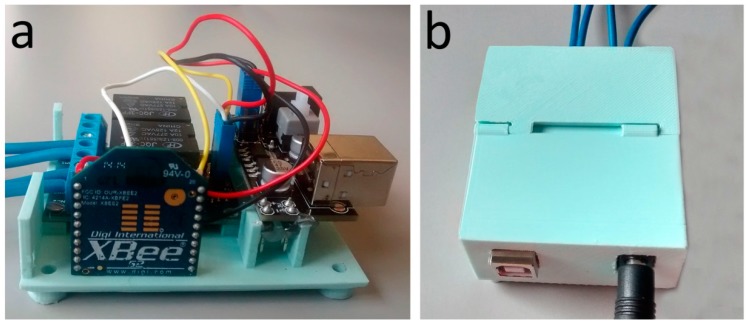
Receiving actuation station: (**a**) internal connection; (**b**) external case.

The receiving actuation station (Figure 4) consists of the following elements:

- XBee S2 module [31];
- 2-Channel relay module [38];
- 5 V/3.3 V power supply module [39].

The XBee S2 module is the core of the actuation station. It receives information from the coordinator module connected to the monitoring station, and sets the digital pin 18 (D2) and 17 (D3) to a high or low value depending on the received information enabling the actuation of the air exchange and illumination systems by means of the two relays.

The cost of the hardware (electronics and 3D printed case) of the receiving actuation station is of about €35.

### 2.3. Data Connection

The overall configuration system provides for the use of two S2 XBee modules (Table 1), that support the ZigBee protocol [40], based on 802.15.4 standard [41]. The XBee module, set as the API coordinator, is connected to the Arduino UNO r3 of the nearable monitoring unit through a specific shield [30]. The XBee module, set as End Device AT, is the core of the actuation unit.

Table 2 shows the structure of the data according to the ZigBee protocol [42].

### 2.4. Control Algorithm

**Figure 5:**
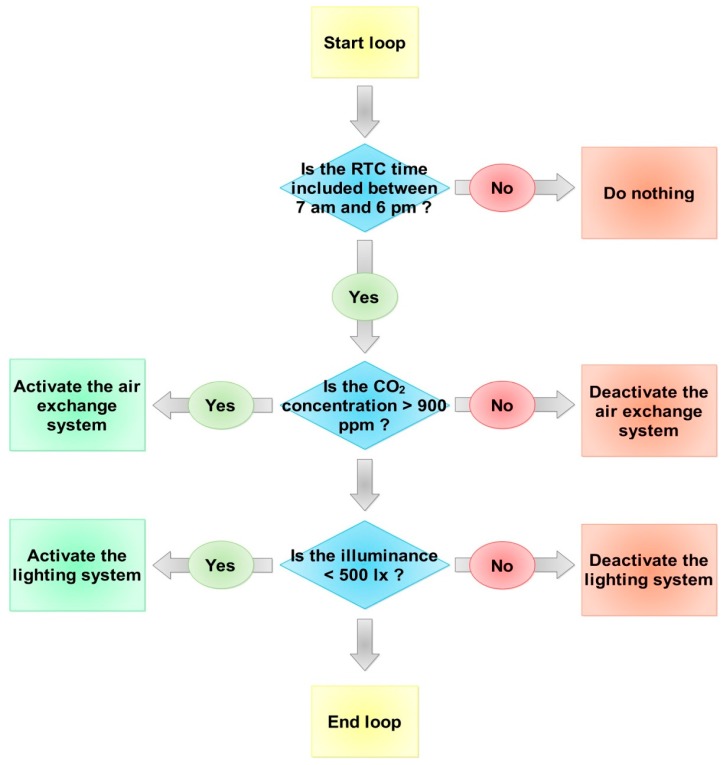
Flow chart of the control logic.

The actuation control logic of the smart lamp allows for management of the lighting and air exchange system in order to optimize both IAQ and ILQ conditions, recording the CO_2_ concentration and the illuminance values. The device is applied during a working day, between 7:00 a.m. and 6:00 p.m. The control system checks the indoor environmental values and performs an actuation in terms of activation/deactivation of the lighting and air exchange systems, if the recorded values deviate from expectations. Figure 5 shows the control logic of the system.

## 3. Case Study and Method of Evaluation of Comfort

### 3.1. Case Study

**Figure 6:**
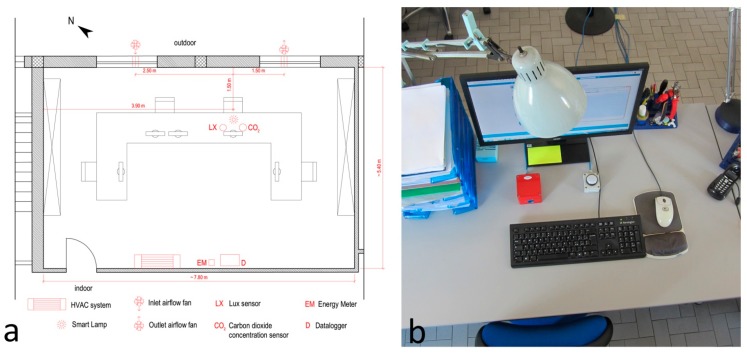
Installation in working conditions: (**a**) plan of the office; (**b**) workstation with smart lamp and sensors.

The system was installed in an office, located on the first and top floor of a building, with an area of about 42 m^2^ (7.81 m × 5.37 m) normally occupied by four workers (Figure 6a). A carbon dioxide (CO_2_) concentration sensor and a lux meter (LX) were installed in the office close to the workplace where the IAQ and ILQ are analyzed (Figure 6b). Both sensors were placed on the desktop next to the nearable and were connected to a data logger (D). The energy meter (EM) ABB OD 1365, connected to the mechanical ventilation and lighting systems, completed the monitoring equipment. Both sensors and energy meters were connected to a data logger (D). The data of the environmental variables were recorded every 10 s then averaged every minute. The consumption data were aggregated and recorded every day. All data were stored on a memory card. The experimentation was carried out from 23 May to 5 June 2016. The experimentation provided two different configurations: in the former, between 23 May and 29 May, the workers could open the windows manually and in the latter, between May 30 and June 05, the automatic control managed the operation of an air exchange system for the air quality control.

The air exchange system consisted of two fans without filters or heat recovery units, mounted on the windows of the office: one introduced fresh air into the office, the other one discharged the exhaust air. Both units were connected to the same relay of the receiving actuation station. The average intake/discharge speed was equal to 2.5 m/s. Considering the diameter of the duct, equal to 0.15 m, its hourly flow rate resulted equal to 159 m^3^/h. This value was determined in compliance with the requirements by the Italian Standard UNI 10339:1995 [43] which for “single or open space” offices, indicates a specific flow rate of air exchange equal to 11 l/s person (equal to 39.6 m^3^/h person). By multiplying this value by the number of people in the office, a flow rate of 158.4 m^3^/h was obtained, fulfilling the requirement.

**Figure 7:**
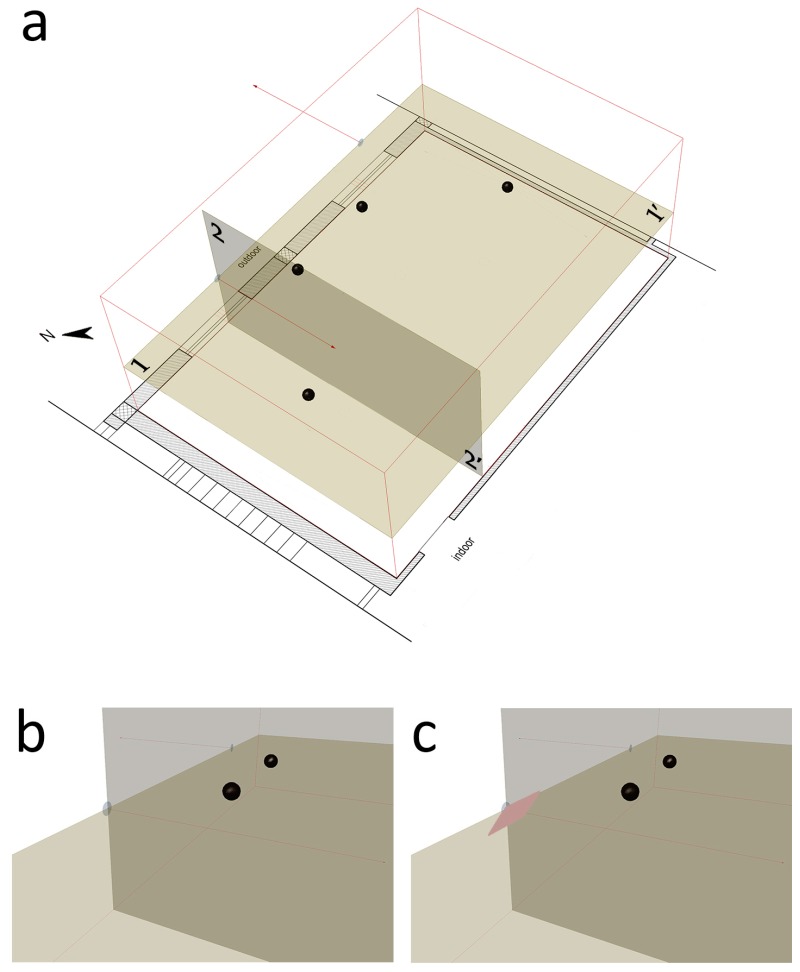
3D representation of the geometric model used for the computational fluid dynamics (CFD) study with reference sections 1-1’ and 2-2’. The black dots identify the positions of the workers: (**a**) office model; (**b**) detail of the starting situation without deflector; (**c**) detail of the optimized situation with the deflector.

The installation of the air exchange system can be a cause of discomfort. The reduced distance between the points where the fans and the workstations were located, could lead to a reduction in terms of indoor comfort due to two effects: an increase of both sound pressure level and air speed. Before the testing period an analysis was made to verify the local discomfort based on user feedback and computational fluid dynamics (CFD) analysis. None of the four workers reported situations of acoustic discomfort. In Figure 7 the black dots are located at a height equal to that of the head of a seated workers, at about 1.3 m. The computational fluid dynamics analysis [44,45] allowed for verification of the air speed levels. It is considered a steady-state solver for incompressible flows with turbulence modeling, known as the simpleFoam solver distributed with OpenFoam.

**Figure 8:**
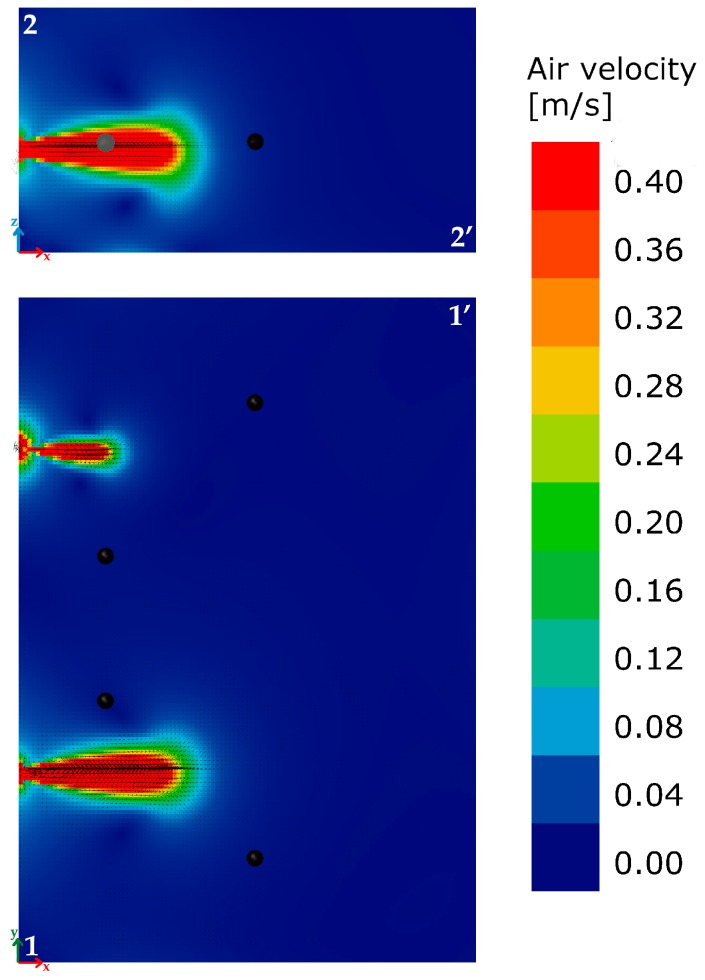
Starting situation: air velocity distribution for sections 1-1’ and 2-2’.

The mesh of CFD model is very dense. This is because the cell size is 0.05 m in all directions in order to capture the smallest detail in the model that is the radius of inlet and outlet flows. Figure 7a takes into account both the horizontal reference section 1-1’ and the vertical section 2-2’ passing through the area of the inlet air flow. Figure 8 shows the trend of the modules of the speed vectors for the two sections. A scale factor of 0.25 has been applied to the velocity vectors.

**Figure 9:**
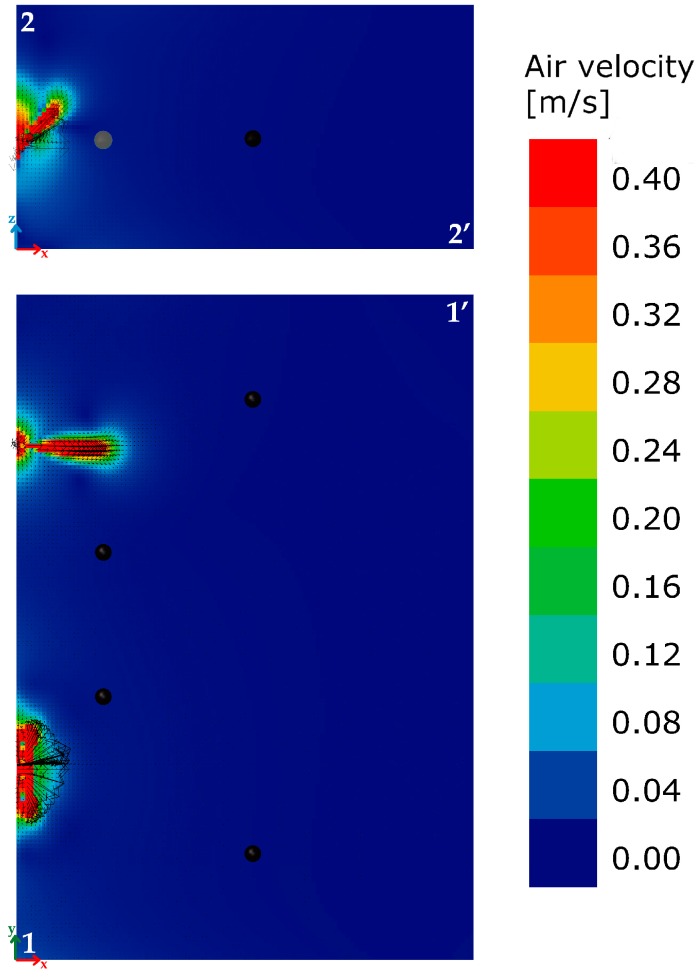
Optimized situation: air velocity distribution for sections 1-1’ and 2-2’.

The EN ISO 7730: 2005 provides the typical values of maximum acceptable air velocity differentiating between summer and winter, and considering the type of environment and specific category defined as a function of the vertical air temperature difference: A < 2 °C, B < 3 °C, C < 4 °C. Assuming a scenario mid-season, with an office as “type of building/space” and the category A, as laid down in the standard, the maximum value of the average air speed equal to 0.12 m/s should be met. An overview of the calculated values (Figure 8) highlights how the average air velocity values are lower than the limit value of 0.12 m/s, with two zone of discomfort. Two working positions are close to the area of discomfort. Given the impossibility of moving the points where the two fans are installed, a deflector of about 60 cm × 40 cm in size, tilted by about 35° as to the vertical plane, was installed close to the lower limit of the inlet air hole (Figure 7c) in order to minimize the possible condition of discomfort due to an excessive air speed. It allowed reduction of the two horizontal components of the velocity vectors, increasing the vertical one. The new simulation (Figure 9) highlighted the area affected by the air movement due to inlet flow decreases. In particular, in section 1-1’, a confined area is affected by the movement of air because the deflector contributes to move up the air towards the upper layers, as is clearly shown in section 2-2’.

The CFD simulations have been verified through iterative convergence of residuals. The positive feedback of the users who had previously highlighted the condition of discomfort due to high air speed values confirms the effectiveness of the solution and allowed for continuation of the experimental analysis.

### 3.2. Influence of Weather Conditions on Monitoring Activity

Outdoor weather conditions must to be considered when IEQ control depends on user control strategies. Several studies [46] have demonstrated the correlation between windows opening and outdoor climate conditions while others [47] have modelled window-opening patterns in relation to the outdoor climate. In this case, the users involved in the study could manage blinds and artificial lighting for ILQ control and windows opening for IAQ control. In relation to the scope, a representative period of the year has been considered to carry out the monitoring activity and to evaluate the performance of the different ILQ and IAQ control systems (manual and automatic). The monitoring periods are selected within spring seasons months (May and June) where:

- heating or cooling plants are turned off;
- weather conditions are quite good: outdoor and indoor temperatures are similar, there is no fog presence but a sufficient quantity of rainy days.

Table 3 reports the main weather data related to the monitoring periods.

### 3.3. ILQ and IAQ Method of Evaluation

The ILQ and IAQ are assessed using the methodologies provided by the technical regulations. In the first case, the level of IAQ is determined considering the concentration of CO_2_ [48]. In this case, the main technical standard is EN 15251:2008 [49] that identifies the optimal values of air exchange for different types and classes of pollution of the buildings, in addition to the concentration threshold values of pollutants in air and in building materials. In particular, the difference in CO_2_ concentration between indoor (CO_2,i_) and outdoor (CO_2,o_) air (Table 4) highlights whether the ventilation strategies of the rooms are correct and whether the air is sufficiently pure.

The standard also defines the typical values of CO_2_ concentration in the outdoor air (CO_2,o_):

- 350 ppm for rural areas;
- 400 ppm for small towns;
- 450 ppm for urban centres.

A reference value of 400 ppm as CO_2_,_o_ and a level of at least II of air quality (Table 4) are considered. Consequently, the CO_2_ concentration limit is fixed at 900 ppm.

**Figure 10:**
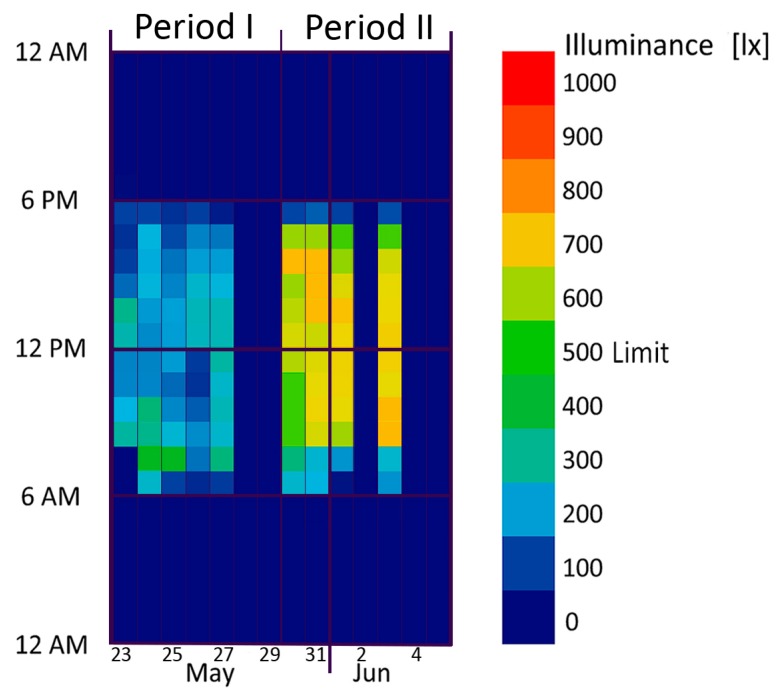
Hourly average data for manual control (period I, 23–29 May) and automatic control (period II, 30 May–5 June): illuminance level.

For ILQ evaluation, the main standard is EN 12464-1:2011 [50] which defines the minimum required levels of illuminance (lux minimum) to carry out specific indoor activities. For writing, reading and data processing, the main activities performed in the office where the experimentation was conducted, the minimum illuminance value admitted is 500 lx (tagged as “limit” in Figure 10).

## 4. Experimentation Results

The levels of ILQ and IAQ and the associated energy consumption for the considered workplaces were assessed. The experimentation lasted 2 weeks and was divided into two periods: period I, between 23 May and 29 May 2016, with manual control of lighting system and air exchange and period II, between 30 May and 5 June 2016 with automatic control provided by the developed system.

### 4.1. ILQ

Figure 10 graphically [44,51,52] represents the hourly average values of illuminance. The periods 28–29 May and 4–5 June correspond to Saturday and Sunday, while June 2 is an Italian national holiday (Italian Republic Day). Considering only the working days and the daily operating time frames (form 9:00 a.m. to 6:00 p.m.) the levels of illumination for the first 5 days of period I are below the minimum level (500 lx, “limit” in Figure 10) required by the national standard for activities related to writing, reading, typing and data processing. In the second case, vice-versa, the daily average value is above the minimum value.

In period I, on the left side, 0% of the hourly average values is higher than the limit of 500 lx between 9:00 a.m. and 5:00 p.m. In period II, on the right side, 100% of the values is greater than the minimum value (Figure 10). These results are strengthened by the fact that the average daily solar radiation is 26% higher during period I (manual control) than period II (automatic control) as can be seen in Table 1.

### 4.2. IAQ

**Figure 11:**
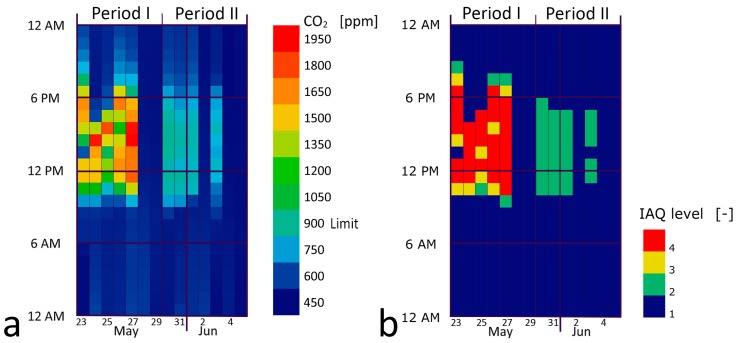
Hourly average data for manual control (period I, 23–29 May) and automatic control (period II, 30 May–5 June): (**a**) CO_2_ concentration; (**b**) indoor air quality level.

Figure 11 shows the hourly average values of the CO_2_ concentration. The dates 28–29 May and 4–5 June correspond to Saturday and Sunday, while 2 June is an Italian national holiday (Italian Republic Day).

The analysis of CO_2_ concentration (Figure 11) shows a marked improvement after the adoption of the nearable control system: in period I, during working hours, hourly average values with maximum values close to 2000 ppm were recorded, in any case higher than the 900 ppm limit (defined in the previous paragraph 3.3), with high range of excursion. In period II, the level of CO_2_ concentration in the air was maintained below the threshold value with small variations over the time.

In analogy to ILQ control assessment, the effectiveness of the automatic control is strengthened by weather conditions. In fact, despite quite similar wind mean velocity values between two periods, in period I (manual control monitoring) the precipitations were much lower than in period II (22.6 mm vs. 118.2 mm) and also rainy days were much different (2/7 vs. 6/7). In addition, the external mean temperature was higher, encouraging users to open the windows for thermal comfort optimization. Although in the first period the weather conditions were optimal to manage manual window opening, the IAQ levels between two periods are very different.

### 4.3. Electrical Consumption

**Figure 12:**
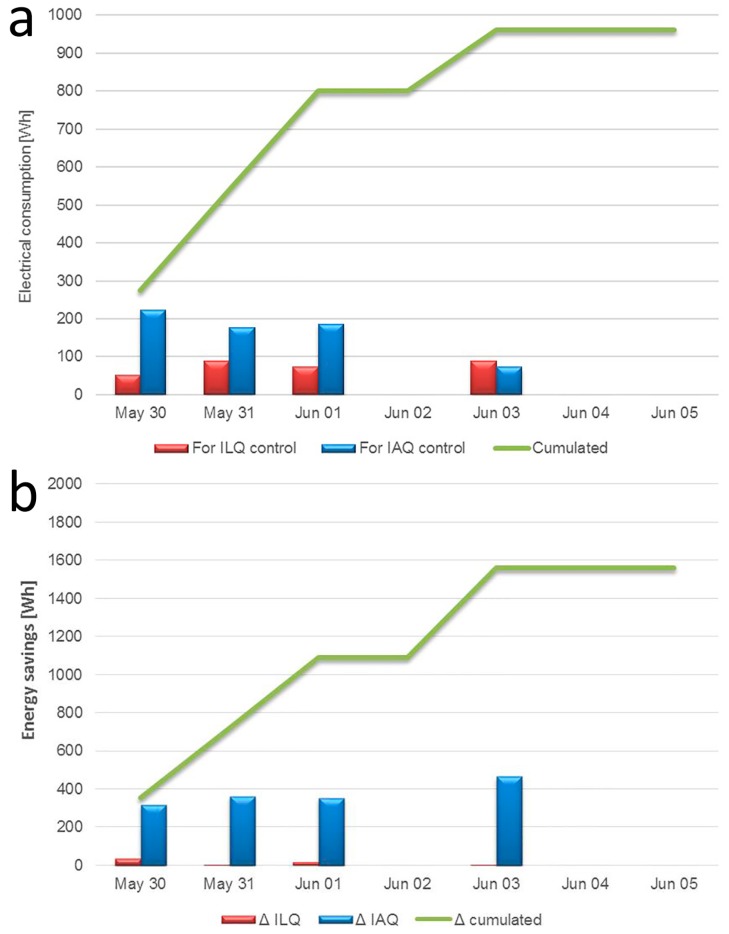
Analysis of consumptions: (**a**) electrical consumptions; (**b**) energy savings. ILQ: indoor lighting quality.

The analysis of the electrical consumption recorded by the energy meter connected to the data logger provides the overall consumption related to the use of this system (Figure 12a). Only period II with automatic control is considered. The daily energy consumption due to lighting is almost constant and below 100 Wh. The consumption related to the air exchange system is almost equal to 200 Wh for the first three days, then on 3 June, when the office was occupied by only one person, the electrical consumption was halved. In Figure 12b the energy savings of the system are reported, considering a comparison with the case when the ventilation and lighting system are run all the time, without a control system. It is clear that the energy saving is due primarily to the air exchanger system control. Over a four-day period of real use, the system allowed a saving of about 1500 Wh.

Considering a typical work week of 5 days, the total energy savings would be of 1.9 kWh that is equal to about 90 kWh for 46 working weeks. Taking into account a price of electricity of €0.18 /kWh an annual saving of about €16/year can be calculated. If a total cost of the both nearable monitoring and receiving actuation stations of €165 is considered, the payback time (without extra costs) would be about 10 years. The payback time could be reduced considering the cheapest CO_2_ concentration sensor. Alternatively, a complete station can be considered that allows optimization of the indoor climate quality and related energy consumption by adding an integrated temperature sensor and an infrared light-emitting diode IR-LED as shown in [18]. The total cost of the system could be €185. The energy saving for a summer seasonal period (approximatively from May to September), would be of about 5 kWh per week that is equal to 100 kWh. The overall annual energy saving could be of 190 kWh/year that is equal to €34.2/year and the payback time (without extra costs) would be slightly above 5 years.

## 5. Conclusions

The developed system demonstrates that a nearable system designed and realized starting from the maker movement philosophy and DIY approach is suitable to ensure good levels of both IAQ and ILQ. The analysis conducted so far demonstrates how it is possible to optimally manage the indoor environmental comfort using some electronics components and a 3D printer. The potential of this basic device is confirmed by tests in real working conditions. The characteristics so far described and analysed allow a wide field of application aimed at improving users’ satisfaction and optimizing energy consumption of buildings. As shown above, the advantages of implementing a system based on a DIY approach with respect to commercial systems are associated with better customization and adaptation options, thus allowing a hacking of a common object, making it capable of carrying out smart operations in order to make the indoor environment more comfortable. In this context, the user is not limited to the passive role of consumer, as he acts as prosumer, actively participating in the various phases of the management and improvement of the environmental quality of the building where he lives. As calculated in the previous paragraph, the solution could be economically favourable if it is considered a complete station able to control ICQ, IAQ and ILQ.

## Figures and Tables

**Table 1:** XBee modules configuration.

ZigBee End Device AT	ZigBee API Coordinator
SH: 0013A200	SH: 0013A200
SL: 40BF9952	SL: 40C143E8
PAN ID 1984	PAN ID 1984
MY: 5ECA	MY: 5ECA
BAUD rate 9600	BAUD rate 9600
DH: 13A200	DH: 0013A200
DL:40C143E8	DL: 40BF9952
D3(17) Digital out, low [4]	
D2(18) Digital out, low [4]	

**Table 2:** Communication data frame structure.

Sequential Number	Value	Purpose
0	7E	Start delimiter
1–2	00–10	Frame length
3	17	Frame type: AT Command
4	00	Frame ID: no reply needed
5–12	000000000000FFFF	Sending broadcast
13–14	FFFE	Destination Network: unknown
15	02	To apply changes
16–17	44–02	Bit mask indicating which pins of the XBee module are enabled for digital output (D2 in this case)
18	05	To set D2 pin to be digital out High
19	70	Checksum

**Table 3:** Weather data for the two configurations—external temperature, solar radiation (the period of diurnal average of solar radiation is from 8 a.m. to 9 p.m.), wind speed, rain.

Period (Configuration)	Environmental Variable	Min	Max	Avg.	Days (Precipitations. >1.0 mm)	Cumulative Precipitations (mm)
I. 23–29 May (manual control)	External temperature (°C)	11.48	32.05	20.88	-	-
	Solar radiation (W/m^2^)	-	946	436	-	-
	Wind speed [m/s]	0.36	2.76	1.48	-	-
	Rain	-	-	-	2/7	22.6
II. 30 May–5 June (automatic control)	External temperature (°C)	14.50	28.44	18.89	-	-
	Solar radiation (W/m^2^)	-	963	345	-	-
	Wind speed (m/s)	0.21	2.60	1.11	-	-
	Rain	-	-	-	6/7	118.2

**Table 4:** Levels of indoor air quality (IAQ) as a function of the indoor–outdoor difference in CO_2_ concentration.

IAQ Level	CO_2,i_–CO_2,o_ Limits
I	CO_2,i_–CO_2,o_ ≤ 350 ppm
II	350 ppm < CO_2,i_–CO_2,o_ ≤ 500 ppm
III	500 ppm < CO_2,i_–CO_2,o_ ≤ 800 ppm
IV	CO_2,i_–CO_2,o_ > 800 ppm
